# Cameron Ulcers: Rare Case of Overt Upper Gastrointestinal Bleed in a Patient with Alcohol Use Disorder

**DOI:** 10.7759/cureus.7644

**Published:** 2020-04-12

**Authors:** Shehriyar Mehershahi, Abhishrut Jog, Diana M Ronderos, Danial Shaikh, Ariyo Ihimoyan

**Affiliations:** 1 Gastroenterology, BronxCare Health System, Bronx, USA; 2 Internal Medicine, BronxCare Health System, Bronx, USA; 3 Medicine/Gastroenterology, BronxCare Health System, Bronx, USA

**Keywords:** cameron lesion, upper gastrointestinal bleed, overt bleed, occult bleed, varices, peptic ulcer disease

## Abstract

Cameron lesion is an uncommon cause of overt upper gastrointestinal bleed (GI bleed). Though hiatal hernia is a well-known entity, Cameron lesions that may occur in them are usually missed during upper endoscopy. Patient with Cameron lesions usually presents as chronic iron deficiency anemia, rarely as acute GI bleed. Multiple other risk factors such as non-steroidal anti-inflammatory drug use, alcohol consumption, gastro-esophageal reflux disease (GERD) may be present concomitantly which makes initial differential diagnosis of Cameron lesions more difficult as seen in our case.

## Introduction

Gastrointestinal bleed (GI bleed) accounts for 350/100,000 hospitalizations annually in the US. Of these, 50% are due to upper GI hemorrhage. The common causes of upper GI bleed in descending order of occurrence are peptic ulcer (38%), esophageal or gastric varices (16%), esophagitis (13%), UGI tract tumor (7%), angioectasia (6%), Mallory Weiss tear (4%), erosions (2%) and Dieulafoy’s lesion (2%). The cause is unknown in 8% of the cases [1].

A Cameron lesion is a rare cause of overt upper GI bleed [2]. It occurs primarily in patients having a hiatal hernia, although not universally [3]. In some patients, it is a cause of iron deficiency anemia due to chronic blood loss, whereas in some it may lead to overt GI bleed. This lesion is often missed on upper GI endoscopy and may go undiagnosed as a result [4]. Knowledge about the existence of this lesion and meticulous search for it in cases of hiatal hernia is necessary for timely diagnosis.

We present one such case of a patient with multiple Cameron lesions with confounding risk factors for other common causes of a GI bleed.

## Case presentation

A 64-year-old male presented to the emergency room with multiple episodes of hematemesis. These episodes had been preceded by heavy binge drinking. He denied any abdominal pain, melena or hematochezia. No previous similar episodes in the past. His past medical history was significant for hypertension, dyslipidaemia, gastroesophageal reflux and ischemic stroke in the past with no residual deficit. Social history was significant for alcohol use of 4-6 shot of vodka daily for past 15 years. He denied illicit drug use or cigarette smoking. There was no significant surgical history. His medications included daily low dose of Aspirin.

On his arrival to ER, blood pressure was 120/70 mmHg, heart rate of 118 beats per minute, respiratory rate of 30 breaths per minute and SaO2 88% at room air. On physical examination, he appeared pale, not in acute distress, with clear and bilaterally equal breath sounds and normal heart sounds. His abdomen was soft and not tender, without any organomegaly. Digital rectal exam revealed dark brown stool.

Hemoglobin at presentation was 5.9 g/dl. Renal function, platelet and white blood cell counts were normal. Prothrombin time, international normalized ratio (INR) and activated partial thromboplastin time values were 13.6, 1.16 and 25.3 seconds, respectively. Total protein and albumin were slightly decreased (5.3 g/dl and 2.3 g/dl, respectively). Aspartate aminotransferase (AST) and alanine aminotransferase (ALT) levels were normal. Ultrasound abdomen showed hepatomegaly of 22.1 cm with diffuse increased echogenicity of the hepatic parenchyma, no splenomegaly. Given history of alcohol use disorder, variceal bleeding was high on the differential. He had no previous esophagogastroduodenoscopy (EGD) performed in past.

**Figure 1:**
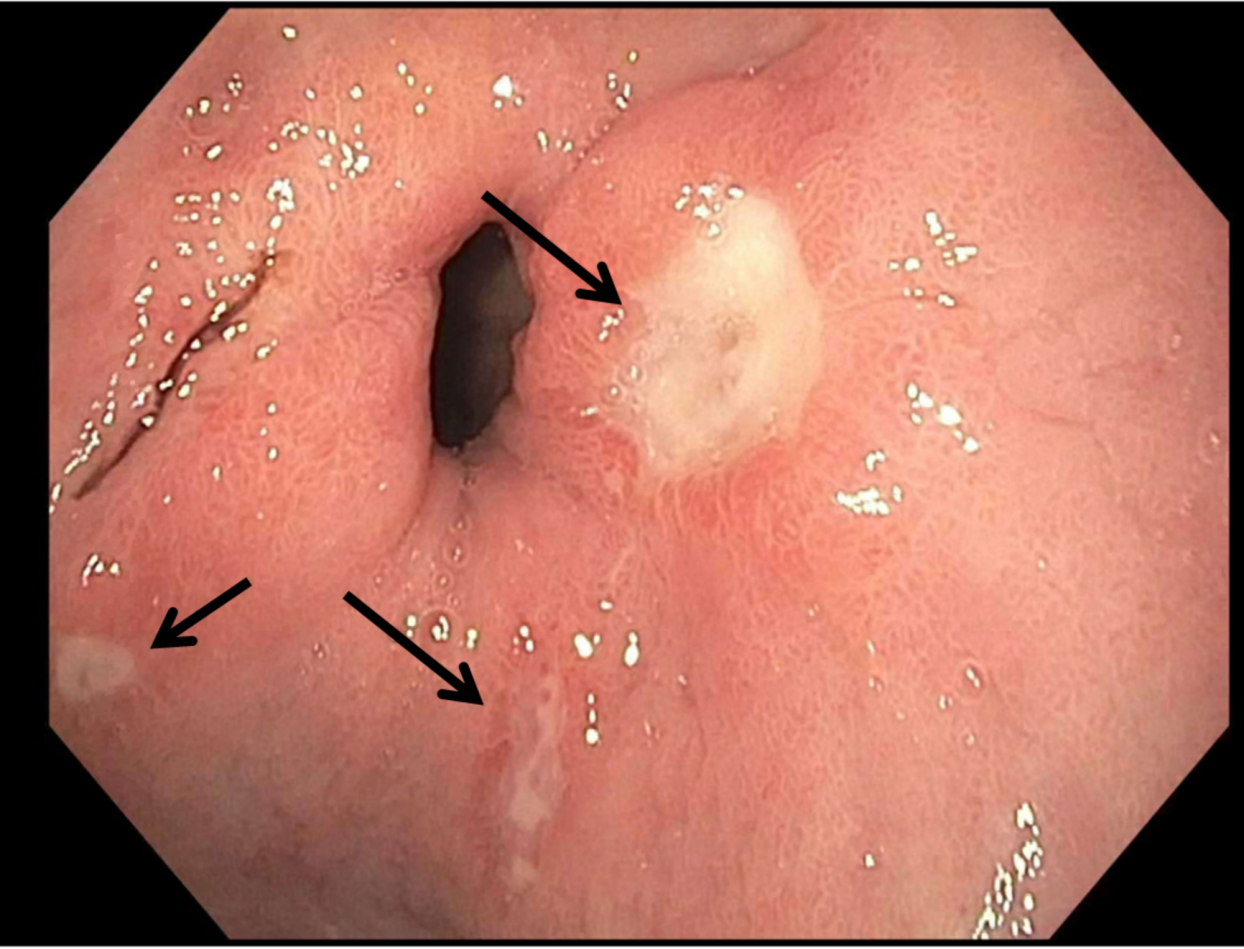
Upper endoscopic image of the Cameron lesions in the hiatal hernia

He was started proton pump inhibitor (PPI) and octreotide. He received two packed red blood transfusion without complications. The patient underwent EGD (Figure 1). It revealed a large hiatal hernia with three Cameron ulcers. The largest lesion was of 10 mm with visible vessel but no active bleeding at its base. The area was successfully injected with 5 mL of a 1:10,000 solution of epinephrine and treated with bipolar cautery. No esophageal or gastric varices noted. No gross lesions were noted in the rest of entire examined stomach, duodenal bulb and in the second portion of the duodenum. The patient was monitored in step down unit and did not have any further drop in hemoglobin. He tolerated diet and was switched to oral PPI. He was discharged on oral PPI and oral iron supplementation to be followed as outpatient.

## Discussion

Cameron lesions were first described in 1986 by Dr Alan Cameron from Mayo Clinic as ‘linear gastric erosions’. They are found in the proximal stomach at the end of a large hiatal hernia near the diaphragmatic pinch [5]. According to Weston, who was one of the first to study the endoscopic characteristics of the Cameron lesions, differentiating ulcers from erosions on the basis of endoscopic appearance and using depth as a criterion is problematic, any ulcer or erosion that occurs at or near the diaphragmatic hiatus is characterized as a Cameron lesion [6].

Overall prevalence of Cameron lesions in all EGD’s is less than 1% [6]. In a study by Camus et al. of 3960 endoscopies, Cameron lesions were found only in 0.6% cases [7]. They are found in 5.2% of all hiatal hernias on EGD [8]. Their prevalence increases with the size of the hiatal hernia. Gray et al. classified hiatal hernia according to size as follows: small (<3 cm), medium (3-4.9 cm) and large (>/=5 cm) [7]. Of all the Cameron lesions they found, 23.2% occurred in small, 32.6% occurred in medium and 44.2% occurred in large hiatal hernias. Weston found similar results [6]. He classified the sizes into small, medium and large as being <2 cm, 2-4.9 cm and >/= 5 cm and found the prevalence of the lesion as 1.6%, 6.4% and 13.7% in each group, respectively [5]. The hiatal hernia in our case was large.

Among obscure GI bleeders the prevalence is around 3.8% [7]. In obscure GI bleeding, the following possibilities exist: the lesion is within the reach of the EGD and was missed, lesion is within the reach of the EGD and is hidden (beneath mucosal folds) or intermittently present (Dieulafoy’s lesion) and lesion is out of the reach of the EGD (in small intestine). Interestingly, 50% cases of obscure GI bleeds are from upper GI lesions that are within the reach of the EGD. The Cameron lesion is one such upper GI lesion (along with gastric antral vascular ectasia and Dieulafoy’s lesion) [1,9].

The classic description of the lesion as given by Cameron is as follows: ‘The erosions were frequently multiple and were usually seen on the crest of an inflamed appearing fold. They were typically white, narrow and elongated with the longitudinal axis corresponding to the longitudinal direction of the gastric mucosal folds’ [5]. The description matches the images provided from the EGD of our patient.

Camus et al. in their case series found that 88% cases have multiple Cameron ulcers, with a median number of three ulcers and a median size of 10 mm [8]. In our case, the patient had three Cameron lesions, the largest being 10 mm. Concomitant endoscopic findings were absent in the study by Moskovitz et al. but were more prominently seen by Cameron and Higgins, and Weston [5,10]. In his study of 50 Cameron lesions on EGD the following concomitant endoscopic findings were noted in descending order of occurrence by Weston: erosive esophagitis (58.9%), peptic stricture (19.6%), Barrett’s esophagus (16%), no findings (16%), erosive gastritis (12.5%), gastric ulcer (12.5%), erosive duodenitis (5.6%), scarred bulb (4%), gastric prolapse (3.6%) [5]. In our case, no other lesion was found in the entirety of the stomach and till 2nd part of the duodenum.

Cameron lesions have a range of manifestations from asymptomatic presentation of an occult GI bleed causing iron deficiency anemia to life-threatening overt GI bleed [2]. Rate of occurrence of overt GI bleed was rare in the study done by Cameron. Presentations were mainly chronic anemia [5]. In subsequent studies the rate of overt GI bleeding has been reported with increasing frequency. In the study done by Weston the following presentations were seen in descending order of occurrence: Acute upper GI bleed (32%), dysphagia (17.8%), GERD symptoms (10.7%), chronic GI bleed (8.9%). It was an incidental finding in 51.8% cases [5]. Camus et al. reported a presence of overt GI bleed in the form of melena, hematemesis, hematochezia in 64% cases and occult GI bleed in 36% cases [8]. In our case, the manifestation was overt GI bleed.

Our patient was a chronic active alcohol user and presented after hours of binge drinking. Additionally, the patient was also on long-term low dose aspirin for a past stroke. Consequently, prior to endoscopy, bleeding from varices, or a peptic ulcer was high on the differential. None of these lesions were seen. The finding of the hiatal hernia prompted the proceduralist toward the possibility of the Cameron lesion. Certain risk factors have been identified in the formation of this lesion. Gray et al., via multivariable logistic regression, showed that in their study, nonsteroidal anti-inflammatory drugs (NSAID) use and a large hiatal hernia were the two statistically significant risk factors for Cameron lesions [7]. The other risk factors studied (male gender, age and absence of PPI use during presentation) were present in our patient, but were not shown to be statistically significant [6]. The average age group of the patients is 70.9 +/- 10 years [7].

Mechanical trauma has been proposed by numerous authors as being the most likely cause of the formation of the Cameron lesion [2,5,11]. Windsor and Collis postulated that three distinct forces act at the neck of the sac during inspiration: (a) The upward and outward forces of negative intrathoracic pressure, (b) the inward impinging force of crural muscle movement and (c) the upward sliding movement of the stomach [12]. Gray et al. suggested that if the Cameron lesion were the result of solely mechanical forces, then the only viable treatment would be surgical [7]. However, the excellent response of this lesion to medical management with oral acid suppressants, leads to the postulation of the co-existence of non-mechanical pathophysiological factors such as gastric acid injury [6]. Other proposed mechanisms are focal ischemia due to diaphragmatic pressure on the herniated sac (Moskovitz), gastric stasis from poor emptying of the pouch, venous outflow and lymphatic obstruction resulting in vascular stasis and edema [5].

Cameron lesions have been treated medically, surgically and rarely endoscopically. Medical management consists of iron supplementation and PPI. Surgical treatment consists of fundoplication [2]. In general, endoscopic management for erosive sources of GI bleed such as the Cameron lesion, is only marginally useful. The outcome is excellent with oral acid suppression, leading to healing of the erosions and normalization of the hemoglobin [12]. Endoscopic management for Cameron ulcer with active bleeding has been described by Lin et al. using band ligation [13,14]. In our case, during endoscopic assessment, no active bleeding was found. Cauterization was done and epinephrine injection was given in the lesion. On stabilization of the patient, he was discharged with oral iron formulation and an oral acid suppressant.

## Conclusions

Cameron lesions are a rare cause of upper GI bleed. The presentation may be acute and overt, instead of chronic and occult as commonly thought. In our case, the patient had an underlying alcohol use disorder which made us focus on possibility of more common diagnosis like variceal bleed and peptic ulcer bleed. Cameron ulcer bleed was not on our differential list. These are also easily missed during upper GI endoscopy due to lack of circumferential involvement and variable degrees of severity (from erosions to ulcerations). This case helps in increasing awareness regarding Cameron ulcers as a possibility of overt upper GI bleed in patient with multiple risk factors like alcohol use disorder and NSAID use.
